# Selection of experimental animals and modeling methods in developmental dysplasia of the hip research

**DOI:** 10.1530/EOR-2025-0006

**Published:** 2025-07-01

**Authors:** Yu Rao, Gonzalo Saiz-Gonzalo, Prateeksha Prateeksha, Anmin Wang, Hongxin Shi, Weiguo Wang, Chuan Li

**Affiliations:** ^1^ Engineering Laboratory of Peptides of Chinese Academy of Sciences, Key Laboratory of Bioactive Peptides of Yunnan Province, Kunming, China; ^2^ Graduate School, Kunming Medical University, Kunming, China; ^3^ APC Microbiome Ireland, Department of Medicine, University College Cork, Cork, Ireland; ^4^ Medical College, Dali University, Dali, China; ^5^ Department of Orthopedics Surgery, China-Japan Friendship Hospital, Beijing, China

**Keywords:** developmental dysplasia of the hip, animal models, evaluation protocols, transgenic animals, genetic manipulation

## Abstract

- Developmental dysplasia of the hip (DDH) is a common neonatal musculoskeletal condition characterized by hip instability and inadequate acetabular coverage. If untreated, it can lead to osteoarthritis, chronic pain, and eventual hip replacement.
- Animal models, including dogs, pigs, sheep, rabbits, rodents, and chickens, are essential tools for studying DDH pathogenesis and testing therapeutic strategies. Larger species closely resemble human anatomy, while smaller species facilitate cost-effective, high-throughput studies and advanced genetic manipulation.
- Key modeling strategies include surgical interventions (e.g. joint dislocation, femoral or pelvic osteotomy), external fixation, and genetic modifications (e.g. gene knockout or lentiviral transduction) to simulate various aspects of DDH and reflect its multifactorial etiology.
- Evaluation techniques such as radiography, microcomputed tomography, MRI, and ultrasound are employed to image bony and cartilaginous structures. Histological and immunohistochemical analyses provide insights into cellular and extracellular matrix changes, while gait assessments evaluate functional deficits and pain-related behaviors.
- Selecting an appropriate animal model requires careful consideration of research objectives, ethical standards, and translational potential. Advances in gene editing technologies (e.g. CRISPR), three-dimensional-printed implants, and *in vivo* imaging are enhancing model fidelity and accelerating the discovery of novel therapies.
- Ongoing innovations in DDH research are expected to bridge gaps in understanding the disease’s etiology and improve long-term outcomes for affected patients through optimized therapeutic interventions.

Developmental dysplasia of the hip (DDH), formerly referred to as congenital dysplasia of the hip, is one of the most common disorders of the neonatal musculoskeletal system, with an estimated incidence of 1–3% (1). Epidemiological studies reveal that approximately one-third of adult patients below 60-years-old who require hip replacement have untreated DDH as the underlying condition (2). Given its high prevalence and potential for leading to severe consequences in adult life, understanding the pathophysiological mechanisms underlying the DDH is crucial for achieving early diagnosis and developing effective, targeted treatment strategies.

Although the etiology of DDH remains unclear, it is widely considered a multifactorial condition arising from both genetic predisposition and mechanical influences. Well-known risk factors include breech presentation, oligohydramnios, swaddling, and a family history of DDH (3). A familial clustering analysis has been reported, with the markedly elevated risk of DDH newborns who have affected siblings (4). Historically, Hippocrates (400 BC) postulated a link between maternal trauma, uterine compression and the development of hip deformities (5). This ancient postulation has been substantiated by modern scientific research. During pregnancy, adverse intrauterine conditions (e.g. small uterine cavity or oligohydramnios) may subject the fetal hip joint to abnormal stress, hindering normal development process of femoral head and acetabular (6). Furthermore, postnatal factors, including restrictive swaddling practices, are also widely recognized as an etiological determinant for hip instability (7).

DDH progresses in a dynamic fashion, often initiated with hip instability subsequently leading to repetitive stretching and injury to surrounding periarticular structures. Eventually, the normal concentric relationship between the femoral head and acetabulum is lost, preventing mutual ‘template’ development and culminating in bony deformities (4, 8). Animal models, therefore, offer an indispensable tool for elucidating the in-depth molecular mechanisms, evaluating novel therapeutic interventions, and conducting preclinical assessment of safety and efficacy. Adherence to ethical guidelines for animal experimentation, including the 3Rs principle – reduction, refinement, and replacement – is essential to minimize animal use and mitigate suffering (9).

In the subsequent sections, we first introduce major animal models for DDH research from a species-based perspective, clarifying each model’s unique attributes. We then discuss evaluation protocols (e.g., imaging, pathology, or gait analysis) employed to assess these models, followed by recommendations on model selection aligned with specific research objectives. This integrative approach highlights each species’ strengths and limitations in DDH studies, providing a comprehensive framework for future investigations.

## Common species and model characteristics in DDH animal models

This section includes species-based overview of often utilized animal models for DDH research. Organizing the content from a species perspective facilitates comparisons of variations in anatomical structures, genetic backgrounds, husbandry costs, and overall feasibility among various animals, which helps researchers in selecting the most appropriate model for their specific goals. For instance, larger animals such as dogs, pigs, and sheep may be more relevant for surgical interventions and biomechanical simulations, while smaller animals such as rabbits, rats, and mice provide advantages in genetic manipulation and high-throughput studies. Furthermore, avian models, such as chickens, are valuable due to their short embryonic development period and the ease of conducting *in ovo* interventions, which yield important insights into early hip morphogenesis. This species-based approach highlights the key characteristics and potential applications of each animal model in the subsequent sections, ultimately enhancing our understanding of DDH pathogenesis and the assessment of potential treatment strategies.

### Dog

#### Key features and applications

Dogs naturally exhibit spontaneous hip dysplasia, which has a strong hereditary component, making them an appealing large-animal model for DDH research (10, 11). The canine acetabulum and femoral head share anatomical and pathological similarities with humans, and the incidence of hip dysplasia varies significantly across breed (0–73.4%) (12, 13). Unlike humans, gender differences in canine dysplasia are less pronounced (14). Since dogs can naturally develop hip dysplasia, selective breeding or utilizing existing dysplastic populations may help researchers observe disease progression that closely mirrors the human condition (15).

#### Typical modeling methods

- **Surgical joint dislocation:** surgery is performed on puppies aged 5–9 weeks, involving the opening of the joint capsule and ligaments, followed by insertion of the gluteus medius tendon into the acetabulum to maintain joint dislocation. Dysplastic changes typically become observable after 3 weeks (16).
- **Femoral rotational osteotomy:** the femoral anteversion angle is increased through femoral rotational osteotomy, which induces anterosuperior acetabular dysplasia and osteoarthritis but does not result in joint dislocation (17).
- **External fixation:** plaster casts are applied to fix the knees of puppies aged 4–6 days, allowing researchers to assess blood supply to the femoral head after dislocation in various fixed positions (18).
- **Dietary manipulation:** puppies are fed high-calorie diets to increase body weight, which elevates the risk of developing hip dysplasia (19).
- **Capsule transection and sutures**: the joint capsule is transversely cut parallel to the femoral neck via a lateral approach, and sutures are applied around the base of the femoral neck to maintain continuous dislocation (20).

Dogs provide a valuable large-animal model for DDH research due to their anatomical resemblance to humans and suitable joint size for reconstructive surgery. The presence of naturally occurring hip dysplasia in certain breeds allows for observation of disease progression under near-physiological conditions. However, their use is constrained by high maintenance costs, the need for extensive facilities, and genetic variability, which can hinder reproducibility and standardization across studies.

### Pig

#### Key features and applications

Pigs exhibit significant similarities with humans regarding cartilage thickness, biomechanical loading, and vascular structures (21, 22). Thus, they are frequently employed to investigate mechanical loads and blood flow perfusion within the hip. The morphological features of the acetabulum, combined with the ample surgical space provided by porcine hips, render pigs well-suited for the development and evaluation of novel DDH surgical techniques and biomaterials (23, 24).

#### Typical modeling methods

- **Frog-leg plaster fixation and allograft transplantation (e.g., meniscus):** applying frog-leg plaster fixation to the hind limbs of neonatal pigs, combined with allogeneic meniscal implants, to induce dysplastic changes in the femoral head and acetabulum within 1 month (25).
- **Tibial intramedullary nails:** placing tibial intramedullary nails with threads at 7 weeks of age to fix the knees and induce hip dysplasia in piglets (26).
- **Acetabular defect creation:** using rongeurs to directly create acetabular defects in adult pigs for surgical or material testing purposes (24).

Pigs are frequently used in DDH research due to their similar cartilage thickness, joint biomechanics, and vascular anatomy compared to humans. Their large joint size is advantageous for surgical simulation and implant testing. Nonetheless, their rapid growth rate, handling difficulty, and high maintenance costs limit their routine use in long-term studies.

### Sheep

#### Key features and applications

Sheep rarely develop DDH spontaneously, making it necessary to induce the condition through experimental methods (27). The relatively large body size of sheep provides an extensive surgical field, closely resembling clinical conditions in humans, which is advantageous for simulating complex orthopedic procedures. Due to their substantial body mass and anatomical features, sheep can be particularly valuable for large-scale biomechanical research and for testing surgical hardware or implants.

#### Typical modeling methods

- **Femoral osteotomy to modify anteversion angle:** A research investigation demonstrated that reducing the femoral anteversion angle via osteotomy in lambs led to acetabular dysplasia (28).

Sheep offer a large operative field and joint size that approximate human anatomy, making them suitable for biomechanical testing and surgical training. However, the absence of spontaneous DDH, coupled with high care requirements and challenges in standardizing induced models, restricts their widespread adoption.

### Rabbit

#### Key features and applications

Rabbits offer a middle ground between large and small laboratory animals, providing sufficient surgical space while remaining relatively cost-effective. Early DDH research frequently employed rabbits. Their use cases are well-documented, offering a rich foundation of existing literature. Although their anatomy differs somewhat from humans, rabbits allow for preliminary surgical and pathological experiments that can inform subsequent studies in larger models.

#### Typical modeling methods

- **Fetal limb extension in pregnant rabbits:** this method involves exteriorizing the fetal hind limbs through a maternal abdominal incision, inserting Kirschner wires perpendicularly, and securing the fetal limb in a fully extended position by suturing the femur and tibia before returning it to the uterus. This approach can induce hip dislocation as early as 2 days post-birth, with a reported dislocation rate of 33% by 1 week (29).
- **External traction devices:** external traction devices applied to the femur, using variable weights and durations of traction, enable precise induction of DDH (30).
- **Restricting lower-limb movement:** restricting lower-limb movement is a commonly used approach for inducing dysplastic changes. Fixing the knees of 3-week-old white rabbits with Kirschner wires for 1 week results in hip dysplasia, which can be partially reversed by selective epiphyseal blockade of the tri-radiate cartilage (TC) at the iliac–sciatic–limb bone (31). However, cutting the distal or proximal hamstrings before knee extension fixation prevents dislocation or subluxation, suggesting that hamstring tension contributes to DDH in this model (32). Materials such as plaster (33, 34) or plastic tubes (35) can also be used to fix rabbit hind limbs for approximately 8 weeks, inducing dysplastic changes (34).
- **Smooth plexiglas flooring:** Some studies have reported a 22% incidence of hip dysplasia in rabbits raised on smooth plexiglas floors (36). However, this method has not been widely adopted for controlled DDH model development.
- **Direct surgical interventions:** direct surgical intervention on hip structures represents another highly efficient method for inducing DDH. For example, placing the excised lateral meniscus of the knee into the acetabulum of 5-week-old New Zealand White rabbits can mimic the inverted labrum configuration observed in DDH (34). Damaging the Y-shaped growth plate in rabbits induces both acetabular dysplasia and hip dislocation, primarily driven by iliac–sciatic injury (37). Surgical excision of posterior acetabulum rim cartilage leads to posterior hip dislocation and acetabular dysplasia (38). Cutting the joint capsule and ligamentum teers can also induce dysplasia, with additional gauze-based hind limb immobilization further exacerbating dysplastic changes (39).

Rabbits strike a balance between size and cost, providing enough joint space for basic surgical procedures and allowing for short-term pathological studies. Yet, their anatomical differences from humans and reliance on artificial induction techniques reduce their applicability in long-term or mechanistic DDH research.

### Rat

#### Key features and applications

Rats are cost-effective, breed quickly, and share notable genetic similarities with humans, facilitating large-scale or longitudinal DDH studies. Their small size and well-established experimental protocols make rats highly versatile for investigating both pathophysiological mechanisms and early intervention strategies.

#### Typical modeling methods

- **Intrauterine limb restriction:** on gestational day 16.5, one fetal hind limb is exteriorized through the uterine wall and sutured to the fetal membrane to restrict movement, while the contralateral limb remains as a control. Despite inducing hip deformities, many offspring recover spontaneously with free activity. The method is limited by surgical complexity, low efficiency, and offspring frailty affecting maternal feeding (40).
- **Hind limb immobilization:** restricting hind limb movement also induces dysplastic changes in rat hips. Fixing the femur and tibia with intramedullary Kirschner wires across the knee in 15-day-old Sprague-Dawley rats for 4 days leads to signs of dislocation (interrupted Shenton’s line) and other pathological changes (41). A simpler approach involves taping newborn rat hind limbs together; after 10 days, acetabular dysplasia and dislocation are observed (42). This taping model can be combined with exposure to fluoride or other risk factors to examine how these factors influence susceptibility to dysplasia (43, 44).

In another study, a 3-week-old rat hind limb was immobilized in extension against a metal rod inside a glass cylinder. After 10 weeks, X-rays showed femoral head and neck dislocation, and the affected femoral head and neck were smaller with a flattened acetabulum. Partial fixation in this model resulted in subluxation rather than full dislocation (45).

- **Surgical destabilization or joint dislocation:** as with larger animals, surgical destabilization or dislocation of the rat hip can mimic the abnormal environment of dislocated joints in DDH, although it differs from human disease onset. One study maintained femoral head dislocation for varying durations to observe its effects on chondrocytes, offering insights applicable to DDH research (46). Another study found that injecting rats with a streptococcal antigen induced synovitis and caused femoral head dislocation and a reduced, flattened acetabulum consistent with DDH. This may have occurred due to joint laxity triggered by the antigen, leading to lateral displacement and eventual dislocation of the femoral head (47). However, this model has not been widely used in further DDH-specific research.

Rats are cost-effective and genetically tractable, making them ideal for high-throughput screening and studies on environmental or mechanical risk factors for DDH. However, their small size and divergent hip biomechanics limit the translation of findings to human conditions.

### Mouse

#### Key features and applications

Mice share a high degree of genetic similarity with humans and are the most used experimental animals. Their small size and low cost, coupled with stable embryonic cells conducive to a range of genetic manipulations, make them crucial model organisms for studying human gene function. Mice are also central to confirming the roles of critical factors in DDH.

**Figure 1:**
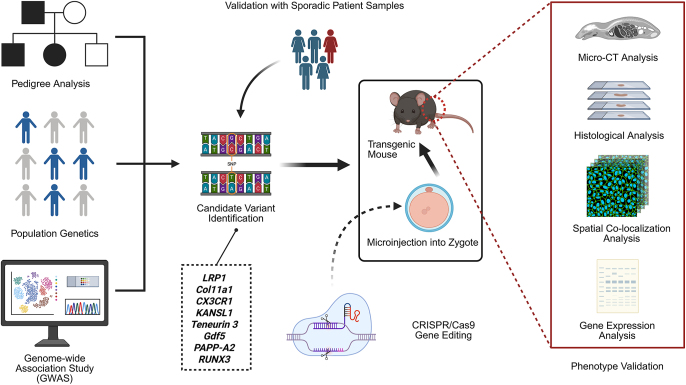
General workflow for generating and validating transgenic mouse models in DDH. Created in https://BioRender.com). The generation of genetically modified mouse models for DDH involves four key steps: (1) identification of candidate pathogenic mutations through pedigree analysis, sequencing of large sporadic populations, or genetic data mining; (2) validation of identified variants in sporadic DDH patient samples; (3) creation of knock-in or knockout mouse models via CRISPR/Cas9 gene editing; and (4) functional validation of mutations and assessment of their impacts on acetabular development using imaging and histopathological analyses.

#### Typical modeling methods

- **Genetic modifications:** genetically modified mouse models have become indispensable tools for elucidating the molecular mechanisms underlying DDH. To clearly illustrate the methodological process and validation strategies involved in establishing transgenic mouse models for DDH research, a workflow diagram is provided in Fig. 1, highlighting key steps from genetic identification to phenotype evaluation. Many candidates’ pathogenic genes were first identified through sequencing analyses of DDH-affected families and subsequently validated in unrelated sporadic cases, offering representative insights into the genetic architecture of the disease. Rodent models provide a critical bridge between human genetic findings and experimental validation of disease mechanisms. In these models, multiple genes associated with DDH pathogenesis have been functionally validated, some of which have also informed preliminary therapeutic strategies. Despite the limited number of studies, these findings offer valuable genetic clues for further investigation. *Lrp1* was identified as a potential susceptibility gene through sequencing of 17 patients from eight DDH families, and validated in 68 patients with severe, dislocated DDH. A knock-in mouse line carrying the *Lrp1*^R1783W^ mutation showed reduced Lrp1 expression. While homozygous deletion proved embryonically lethal, heterozygous knockout (*Lrp1*^+/−^) and knock-in mice exhibited DDH-like phenotypes, characterized by impaired chondrocyte autophagy and disrupted Y-cartilage differentiation. These alterations were attributed to limited mesenchymal stem cell differentiation and β-catenin accumulation following autophagy inhibition. Treatment with the β-catenin inhibitor BUN partially reversed the dysplastic changes *in vivo* (48). *Kansl1* mutations were discovered through whole-exome sequencing of 200 unrelated Han Chinese patients with DDH. Kansl1-mutant mice exhibited decreased and irregularly distributed chondrocytes, likely due to impaired differentiation capacity of mutant stem cells (49). *Col11a1*, identified via GWAS and validated in a cohort of 218 cases and 360 controls, was associated with abnormal cartilage metabolism. *Col11a1* knockout mice showed early-onset and aggravated osteoarthritis-like changes, possibly through a HIF1α-mediated glycolytic–OXPHOS imbalance. Based on these findings, *Col11a1*-overexpressing organoids were developed as a potential therapeutic strategy for DDH (50). *GDF5* is a gene implicated in both DDH and osteoarthritis. In BALB/cJ bp3J mice (Jackson Laboratory, USA), homozygous mutants (bp/bp) exhibited femoral shortening, reduced femoral head and neck size, compromised hip joint stability, and increased risk of dislocation. Notably, crossbreeding with downstream BAC transgenic mice rescued acetabular development in offspring (51). *TENM3* mutations were discovered through sequencing of a three-generation family (15 members) and validated in 11 unrelated DDH cases. Using the *Prrx1*-Cre driver line (B6.Cg-Tg(*Prrx1*-cre) ^1Cjt/J^), the generated mutant mice showed delayed acetabular development on the left side, likely due to increased MMP13 expression, leading to extracellular matrix degradation and inhibited cellular differentiation (52). *Runx3*-floxed knockout mice (*Runx3*^loxP/loxP^) developed severe and irregular DDH-like phenotypes, including a prominent cam deformity at the femoral neck, elevated acetabular index, and shallow acetabulum (53). *CX3CR1* mutations were identified from a three-generation, 12-member DDH family and further analyzed via linkage study involving a four-generation, 72-member pedigree. *CX3CR1* knockout mice (C57BL/6NTac-[KO]*CX3CR1*) exhibited enlarged acetabula disproportionate to femoral head size, with reduced chondrocyte numbers and matrix. Embryonic stem cell differentiation experiments further confirmed impaired cartilage formation (54).
- **Local interventions:** beyond whole-organism genetic modification, viral or pharmacological interventions targeting local tissues can also produce dysplastic hips. For example, subcutaneous injection of 0.1 mL pcDNA3.1-*WNT1* or si-*WNT1* cell suspensions in mice produces acetabular dysplasia at the injection site after 5 weeks (55). Injecting 50 μL Cas9/PAPP-A2 sgRNA lentivirus around the mouse trochanter can induce acetabular dysplasia (56) In CD-1 neonatal mice (day 1 after birth), intramuscular injection of 0.15–0.2 U botulinum toxin A dissolved in saline – twice per week until weaning, then once weekly thereafter – led to hip dysplasia on the injected side by week 4 (57).
- **Surgical instability protocols:** studies have also described surgical protocols that tailor different levels of instability in the mouse hip (58). Operating on 3-week-old C57BL/6 mice, with or without iliacus/iliopsoas tendon release, capsulotomy, transection of the ligamentum teers, or femoral head resection, leads to varying instability. Mild instability does not alter hip morphology; moderate instability causes lateralization of the femur without complete dislocation; and severe instability results in frank dislocation and the formation of a false acetabulum. Femoral head resection reduces the joint space volume (58).

Mice are indispensable for genetic research in DDH due to their ease of transgenic manipulation, short reproductive cycles, and extensive molecular toolkits. Despite this, their extremely small size and anatomical differences pose challenges for surgical modeling and reduce their relevance for biomechanical studies.

### Chicken

#### Key features and applications

Chicken embryos develop within a relatively brief period and outside the maternal body, allowing direct manipulation of the embryonic environment. Relevance to Developmental Disease Research: the ease of accessing and altering chicken embryos has made them a popular model for investigating early developmental processes, including skeletal and joint formation.

#### Typical modeling methods

- **Decamethonium bromide-induced contractural dislocation:** one method uses decamethonium bromide (a neuromuscular blocking agent that induces rigid paralysis) to model contractural dislocation in chicken embryos. Administering the drug via injection into embryos on day 4 of incubation leads to hip dysplasia, affecting both the proximal femur and the acetabulum by day 9 (59).
- **Amniotic fluid aspiration:** another technique involves aspirating amniotic fluid from chicken embryos to induce joint dysplasia; controlling the volume and timing of fluid aspiration can clarify correlations between the severity of dysplasia and the degree of oligohydramnios (60).

Chicken embryos offer a unique opportunity to study early skeletal development and intrauterine-like conditions due to their external embryogenesis and ease of manipulation. Still, their substantial evolutionary distance from mammals and limited genetic tools reduce their translational value in modeling postnatal hip pathology.

### Others

In addition to dogs, many other animal species spontaneously develop hip dysplasia, including cats (61, 62), woodchucks (63), koalas (*Phascolarctos cinereus*) (64), wolves (65), alpacas (66) and roe deer (67). Interestingly, saber-toothed tigers may also have experienced acetabular dysplasia; unfortunately, they cannot be used for contemporary DDH research (68). It is conceivable that researchers might identify spontaneous DDH in other species whose joint structures and mechanical properties more closely resemble those of humans, offering promising directions for future studies.

**Figure 2:**
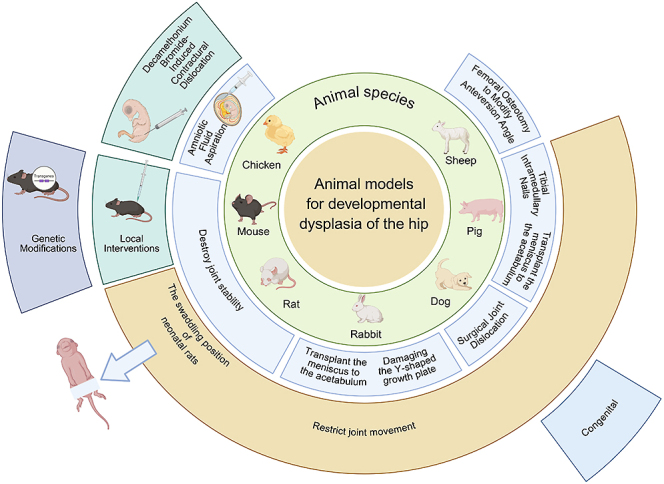
The schematic diagram of common animal models for developmental hip dysplasia (created in https://BioRender.com).

This section offers an overview of the commonly used animal models in DDH research, emphasizing their key characteristics (Table 1, Fig. 2). Since each species varies significantly in body size, genetic background, mechanical loading, and maintenance costs; it is crucial for researchers to choose the model that most accurately reflects the pathological processes of human DDH, tailored to their specific research goals and available resources. In addition, implementing a well-structured evaluation protocol is vital for objectively assessing skeletal and joint changes in each model and for determining the effectiveness of potential interventions, which ultimately aids in clinical translation. In the subsequent discussion, we will concentrate on various evaluation strategies – such as imaging, pathological, and biomechanical assessments – to provide a comprehensive framework for understanding the pathological features of DDH models and for evaluating therapeutic outcomes.

**Table 1:** Comparative overview of common animal models for developmental dysplasia of the hip (DDH)

Animal models	Common modeling methods	Pros	Cons	Typical application scenarios
Large animal models		Closely resemble human anatomy and joint biomechanics. Their large size supports clinical-like orthopedic procedures, making them suitable for implant testing and surgical training. Dogs may naturally develop DDH, enhancing their relevance, while sheep are widely used due to favorable joint structure and load-bearing capacity	Spontaneous DDH is rare, surgical induction is often required, and their use is limited by high cost, complex housing needs, and long-term care demands	
Dog	• Spontaneous genetic/close inbreeding (10, 11, 15) • Surgical interventions (capsulotomy, rotational osteotomy, tendon transposition, etc.) (16, 17, 20) • External fixation (casts, splints) (18) • High-calorie feeding (19)			• Studies on the natural development and pathogenesis of DDH • Simulation of extensive surgical procedures and joint replacements • Long-term mechanical and safety evaluation of biomaterials or implants
Pig	• Frog-leg cast fixation in neonatal piglet allograft transplantation (e.g. meniscus) to induce deformities (25) • Surgical creation of acetabular defects or subluxation (24, 26)			• Large-animal surgical models (e.g. pelvic osteotomies, implant testing). • Studies on blood flow dynamics, perfusion, and biomechanical simulations • Research on bone repair and regenerative materials
Sheep	• Very low incidence of spontaneous DDH (27) • Adjusting femoral anteversion angle (femoral osteotomy) to induce acetabular dysplasia (28)			• Simulation of orthopedic surgery • Large-scale biomechanical experiments • Long-term follow-up studies (e.g. joint replacement or bone healing mechanisms)
Moderate-size models				
Rabbit	• External traction or fixation (casts, plastic tubes, Kirschner wires, etc.) (30, 31, 32, 33, 34, 35) • Surgical intervention (capsule incision, damaging Y-shaped cartilage) (37) • Tissue transplantation (e.g. meniscus to simulate inverted labrum) (34)	Balance surgical feasibility with lower cost. Adequate joint space and extensive literature support their use in early DDH pathogenesis and surgical correction studies	Biomechanical differences from humans, reliance on induced dysplasia, and limited capacity to replicate disease progression restrict their translational value	• Mid-sized experimental models • Pathological studies (cartilage, trabecular bone) • Preliminary validation of surgical procedures or pharmacological interventions
Small animal models				
Rat	• Intrauterine fetal limb immobilization (40) • Postnatal hind-limb immobilization (cast, tape) (41, 42, 43, 44) • Surgical destabilization or dislocation (46, 47)	Inexpensive, easy to breed, and ideal for genetic studies due to short reproductive cycles and mature gene-editing tools. They are widely used to validate pathogenic genes and therapeutic targets	Applicability in complex surgeries and implant testing Significant anatomical differences, small surgical fields, and rapid disease progression limit their	• Genetic/molecular mechanism studies • Initial assessments of pharmaceuticals and rehabilitation • Early-stage biomechanical and cartilage damage experiments
Mouse	• Gene knockout/transgenic approaches (*Lrp1^+/-^*, *GDF5* KO, etc.) (48, 49, 50, 51, 52, 53, 54) • Viral vector or pharmacological injections (55, 56) • Surgical interventions (capsulotomy, femoral head resection) (57)			• Gene function and molecular pathway research • High-throughput drug screening • Early pathological and developmental studies of the hip joint
Avian models				
Chicken	• Neuromuscular blockade injection on day 4 of incubation (decamethonium bromide) (58) • Amniotic fluid aspiration to mimic oligohydramnios (59)	Chicken embryos, allow easy *in ovo* access for developmental studies. Their short embryonic period and manipulability support early pharmacological and mechanical research	Their evolutionary distance from mammals and lack of weight-bearing function confine their use to early-stage developmental studies	• Investigating early hip development • Examining intrauterine-like conditions (e.g. oligohydramnios) on DDH • Basic developmental biology studies

## Evaluation protocols for DDH animal models

Having introduced the core animal models in a species-based manner, we now turn to the common evaluation protocols employed in DDH studies. These methods include a variety of imaging and pathological assessments and functional analyses.

### Radiograph

Current methods for evaluating DDH models primarily include imaging and pathological analysis, with X-ray imaging being the most used approach. Because canine hip dysplasia is relatively common, it has standardized diagnostic criteria (69). Physical examination and imaging help evaluate dysplastic changes. Pain, restricted range of motion, and abnormal gait or behavior may signal hip dysplasia (10). The Barlow and Ortolani maneuvers, well-known in human DDH screening, can also be applied to dogs, but anesthetic assistance is often needed to avoid false negatives.

Radiograph is the gold standard for diagnosing and assessing hip development in dogs, typically via an anteroposterior view of the pelvis. Measurements such as the center-edge (CE) angle and the Norberg angle (NA) are used to assess femoral head coverage by the acetabulum. Breed-specific diagnostic thresholds has been established; for instance, CE angle below 20.8° indicates dysplasia in German Shepherds (69), while below 27° in Labrador Retrievers (70).

### High-resolution microCT

For other animal species, no uniform radiographic criteria exist. Researchers generally rely on anatomic landmarks and imaging markers, including Shenton’s line and Perkins quadrant, to assess joint integrity (24, 28, 34, 42). With advancements in imaging technology, high-resolution microcomputed tomography (microCT) has become an increasingly popular tool for evaluating DDH models (54, 71). MicroCT allows multidirectional, multiplane observation of hip structures and supports quantitative bone analysis. It also facilitates the study of pathological and physiological processes such as osteoarthritis and bone remodeling by examining changes in local trabecular microarchitecture (72).

### MRI and ultrasound

For models without fully ossified hip joints, X-rays are insufficient to capture the femoral head–acetabulum relationship. While ultrasound is essential for early DDH screening in humans, it is less commonly employed in animal studies. Complementary ultrasound evaluations are sometimes recommended for canine hip screening to assess soft tissue structures (73). However, in dogs, ossification begins around 8 weeks of age, and acoustic shadowing from bone hinders ultrasound imaging (10). Rodent hip anatomy differs from humans, with the greater trochanter possibly blocking the acoustic window. Nonetheless, arthrography, MRI, and ultrasound remain potential modalities for future research.

### Pathological and histological analyses

Pathological assessment, widely employed in research, allows direct observation of multilevel tissue changes (42, 54). However, current analyses primarily focus on gross and histological examinations of bone and cartilage, while soft tissue structures, such as the joint capsule, ligaments, and the labrum, often receive less attention. Expanding the scope of pathological studies to include these structures could provide a more comprehensive understanding of DDH pathophysiology.

### Gait analysis

Gait analysis is another way to evaluate musculoskeletal disorders in experimental animals, indirectly reflecting limb pain, restricted motion, and biomechanical changes (74, 75). One study using a Digi Gait system revealed gait abnormalities in DDH mice (54). However, in quadrupeds, the correlation between gait anomalies and DDH severity may be weaker than in humans (10, 69), and differences in weight-bearing directions complicate comparisons between canine DDH and human clinical presentations (10). As a result, gait analysis remains only an auxiliary indicator rather than a diagnostic standard for DDH or its severity.

This section offers a thorough overview of the common evaluation methods utilized in animal models of DDH, including various imaging techniques such as X-ray, microCT, MRI, and ultrasound, alongside pathological examinations such as histology and immunohistochemistry, and gait analyses (Table 2). Each method presents unique advantages and limitations; for instance, X-ray is particularly effective for initial diagnoses and assessing bone structure, while MRI excels in visualizing soft tissues. Histological analysis provides detailed insights into microscopic changes within the joint, although it is restricted to endpoint evaluations. Researchers must carefully consider these factors in relation to their specific research objectives and available resources to achieve the most meaningful results. In the following section, we will explore how to effectively select or combine these evaluation strategies based on study goals, the chosen animal model, and overall feasibility, thus enhancing both scientific rigor and practical application.

**Table 2:** Key evaluation methods and considerations for DDH animal models.

Method	Main purpose	Advantages	Limitations
X-ray radiography	To assess hip joint alignment (e.g. Shenton’s line, CE angle) and bone morphology	Cost-effective, easy to perform, and the ‘gold standard’ for initial bone assessment	Limited information on soft tissues; less accurate for unossified structures (e.g. neonatal models)
Anteroposterior view			
Lateral or specialized views			
MicroCT	Enables detailed three-dimensional visualization of bone microarchitecture and quantitative analysis of trabecular changes	Provides high-resolution, multi-planar reconstructions; valuable for studying osteoarthritis progression and bone remodeling	Requires euthanasia or access to specialized *in vivo* microCT equipment; associated with high costs and concerns about radiation exposure
High-resolution three-dimensional imaging			
MRI and ultrasound	MRI provides superior soft tissue contrast; ultrasound is useful for evaluating developmental stages (especially in neonates)	MRI gives detailed soft tissue imaging (joint capsule, labrum); ultrasound is noninvasive, real-time, and particularly helpful before ossification	MRI is expensive and requires sedation/anesthesia; ultrasound may have limited acoustic windows in certain species (e.g. rodents)
MRI for soft tissue			
Ultrasound for early screening			
Pathological and histological analysis	Direct observation of cartilage, bone, and other joint structures at the tissue and cellular levels to confirm dysplastic changes	Allows detailed characterization of structural changes and molecular markers; can validate imaging findings	Invasive, endpoint analysis; cannot assess dynamic changes in a living subject
Gross morphology			
Immunohistochemistry			
Histology (e.g. H&E, Safranin O)			
Gait analysis	Indirectly reflects musculoskeletal function, pain, and range-of-motion limitations, offering quantitative parameters (step length, stance time, etc.)	Noninvasive, provides functional outcomes, and can detect subtle lameness or asymmetry	Data interpretation can be challenging in quadrupeds due to breed- or species-specific gait patterns, which may influence results
Treadmill (e.g. DigiGait™)			
Motion capture systems			

## Selecting a DDH animal model

Model selection must first consider whether it can address the specific research question. Practicality, cost, available resources and facilities, and the researchers’ expertise are also crucial factors. DDH animal models span diverse species, from large animals to rodents, each with unique advantages and drawbacks.

Large-animal models allow complex surgical interventions to test therapeutic techniques or medical devices. Their physiology, anatomy, and hemodynamics more closely mirror those of humans, enabling robust clinical assessments. Small-animal models, on the other hand, facilitate cost-effective, high-throughput studies, and their short breeding and life cycles allow for comprehensive disease observations over the lifespan. Rodents (particularly mice) offer the added advantage of easy transgenic manipulation – an essential tool for dissecting gene function in DDH. However, the anatomical and biomechanical differences between rodent hips and human hips can limit the direct translational value.

Current DDH research focuses on the disease’s etiology, risk factors, natural history, and developing effective treatments. Etiological studies, including genetic and mutational analyses, frequently use mice due to the difficulties associated with generating large-animal transgenic models. Numerous transgenic or knockdown/knockout rodent models have been developed to investigate the functional relationships between specific genes or proteins and DDH (48, 49, 51, 52, 54, 55). Once preliminary evidence highlights the importance of a particular gene or molecule, transgenic approaches can be employed to confirm its role. As a less resource-intensive alternative, local injection of lentiviral vectors to modify key molecular expression *in situ* provides another option for exploring DDH mechanisms (56). Another strategy is ‘reverse’ exploration of spontaneous canine dysplasia, although the cost and logistical challenges are non-trivial.

Among the various risk factors for DDH, including swaddling, breech position, reduced amniotic fluid, and trace element imbalances (4), the swaddling or hind limb immobilization model is the most used. This approach, especially popular in rats and rabbits, offers simplicity, time efficiency, and high success rates. Therefore, rat swaddling model is often considered as a first-line model for investigating DDH. Such models can also be combined with other risk factors, such as mineral imbalances, for more complex disease assessments, and may serve as a basis for testing candidate therapeutic agents. Surgical modification of the intrauterine environment in pregnant mammals remains technically challenging and inefficient, with many newborns failing to exhibit a stable dysplastic phenotype. Egg-laying species, such as chickens, offer a more tractable option for modeling oligohydramnios due to their external embryonic development. However, the significant anatomical and physiological differences between chickens and humans should be carefully considered when interpreting results.

DDH’s natural history typically involves joint instability, subluxation, dislocation, and osteoarthritis, although some patients do not present with outright dislocation (4). Cartilage damage, inverted labrum, and femoral neck deformities are very common, warranting further study of the roles of mechanical and structural abnormalities. For research focusing on these structural consequences rather than on etiology, low-cost, short-lifespan models such as rats and rabbits are suitable. Techniques such as swaddling, casting, or knee immobilization suffice. Where a single structure’s role is under scrutiny (e.g., cutting specific stabilizing structures in mice or damaging targeted regions of the Y-cartilage in rabbits), more precise surgical interventions are appropriate. If dynamic, complex assessments – such as hemodynamic evaluations or more extensive surgical manipulations – are required, large animals (dogs or pigs) become preferable.

Currently, no pharmacological treatment for DDH exists; therapy involves modifying mechanical and anatomical conditions of the hip (4). Early-stage interventions aim to reduce dislocation and maintain alignment, whereas mid-to-late stages generally require additional surgeries (4). Consequently, research on new treatments often calls for large-animal models (e.g., dogs or pigs) to evaluate acetabular osteotomy, joint replacement, or other invasive procedures over an extended period. These models are the gold standard for assessing procedure-related outcomes and complications.

**Figure 3:**
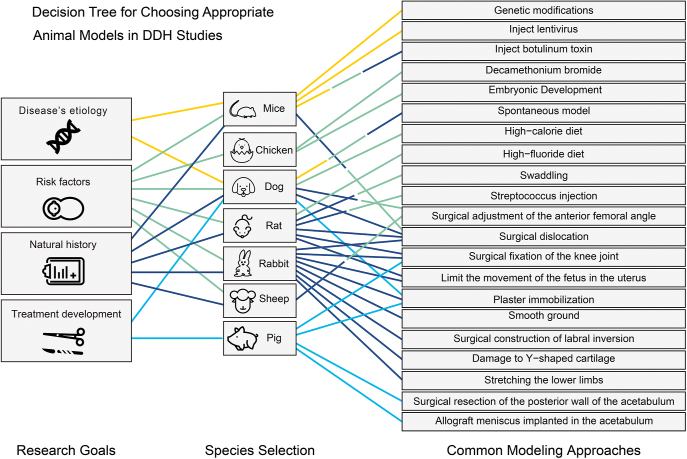
Decision tree for choosing appropriate animal models in DDH studies. This schematic uses different colors to distinguish between research objectives such as investigating disease etiology, risk factors, natural history, and treatment development. For each objective, lines of the same color connect the applicable animal species and modeling approaches, indicating suitable combinations. When a single modeling method connects to multiple objectives, it suggests cross-applicability across different research goals.

It is important to emphasize that every DDH model possesses unique features (Fig. 3). Researchers must balance their objectives with resource constraints when choosing an animal model.

## Ethical considerations in animal models

The use of animal models in DDH research must adhere to internationally recognized ethical standards. Central to these is the principle of the 3Rs – replacement, reduction, and refinement – as articulated in legal frameworks such as the EU Directive 2010/63/EU, which emphasizes ethical responsibility in animal research. Some guidelines also include a fourth ‘R’ for responsibility, underscoring the scientific community’s duty to ensure the humane treatment of research animals (76, 77).

All studies involving animals should obtain prior approval from an Institutional Animal Care and Use Committee (IACUC) or equivalent ethics body (78). Adherence to the ARRIVE guidelines is encouraged to improve transparency and reproducibility, and to reduce unnecessary duplication of animal studies (79).

Practical implementation of these principles includes selecting the least sentient or simplest appropriate species using proper experimental design (e.g. power analysis) to determine the minimum number of animals needed, and refining procedures to minimize pain and distress – including the use of anesthesia, analgesia, and clearly defined humane endpoints. Postoperative care must be standardized and species-specific housing and enrichment should be provided.

## Conclusion

Although gait differences between quadrupeds and humans limit direct biomechanical extrapolation, particularly in studies involving load-bearing mechanics, these differences do not preclude the relevance of animal models in DDH research. Quadrupeds begin locomotion shortly after birth, whereas humans develop upright gait gradually over months, contributing to divergent mechanical environments during hip joint development. However, at the histopathological and cellular levels, many animal models replicate human DDH phenotypes with high fidelity, supporting their continued utility in mechanistic and biomarker studies.

While bipedal mammals such as non-human primates offer the closest anatomical and biomechanical parallels to humans, their high cost, long reproductive cycles, and ethical constraints hinder routine application. Avian models such as chicken embryos, although phylogenetically distant, provide unique advantages for studying early morphogenesis due to their upright embryonic posture and external development. Some study using a chicken embryo model highlights the feasibility of modeling intrauterine stress during joint formation. Building on this, developing oligohydramnios models in mammalian species could offer a more physiologically relevant system for investigating how reduced amniotic fluid impacts hip development. Such models may bridge the gap between simplified avian systems and the complex maternal–fetal environment in humans.

Rodents, despite their quadrupedal gait, remain essential due to genetic tractability and conserved developmental pathways. Many DDH-associated genes, including CX3CR1, LRP1 and so on, exhibit strong cross-species conservation, enabling gene-edited models to simulate human-like disease phenotypes. These systems are now widely used to explore the genetic and molecular basis of DDH.

This review outlines the comparative characteristics of large animals (dogs, pigs and sheep), mid-sized models (rabbits), and small or avian models (rodents and chickens). Each offers distinct advantages and limitations in anatomical relevance, cost, scalability, and technical accessibility. No single model fully replicates the multifactorial and heterogeneous nature of human DDH. Therefore, model selection should align closely with specific research goals – whether focused on genetic mechanisms, biomechanical evaluations, or therapeutic validation.

Future directions include refining model induction strategies, improving imaging and analytical tools, and exploring emerging technologies such as CRISPR-based gene editing and three-dimensional-printed implants. Together, these approaches will enhance the translational relevance of preclinical models and accelerate the development of improved diagnostics and treatments for DDH.

## ICMJE Statement of Interest

The authors declare that there is no conflict of interest that could be perceived as prejudicing the impartiality of the work reported.

## Funding Statement

This work was supported by the Technical innovation Talents Training Object Project Chuan Li (Grant No. 202005AD160146); National High Level Hospital Clinical Research Funding, 2023-NHLHCRF-YXHZ-ZRZD-04.

## Author contribution statement

YR helped with conceptualization and designing, and writing of the original draft. GSG helped in writing of the review and editing, proofreading and formatting. HXS and AMW helped in data collection. WGW helped in project administration. CL helped in conceptualization and designing and funding acquisition.

## References

[1] Guille JT, Pizzutillo PD & MacEwen GD. Development dysplasia of the hip from birth to six months. J Am Acad Orthop Surg 2000 8 232–242. (10.5435/00124635-200007000-00004) 10951112

[2] Bache CE, Clegg J & Herron M. Risk factors for developmental dysplasia of the hip: ultrasonographic findings in the neonatal period. J Pediatr Orthopedics B 2002 11 212–218. (10.1097/01202412-200207000-00004) 12089497

[3] Xiao H, Tang Y & Su Y. Risk factors of developmental dysplasia of the hip in a single clinical center. Sci Rep 2022 12 19461. (10.1038/s41598-022-24025-8) 36376447 PMC9663425

[4] Sewell MD, Rosendahl K & Eastwood DM. Developmental dysplasia of the hip. BMJ 2009 339 b4454. (10.1136/bmj.b4454) 19934187

[5] Woodacre T, Ball T & Cox P. Epidemiology of developmental dysplasia of the hip within the UK: refining the risk factors. J Child Orthop 2016 10 633–642. (10.1007/s11832-016-0798-5) 27866316 PMC5145848

[6] Harsanyi S, Zamborsky R, Krajciova L, et al. Developmental dysplasia of the hip: a review of etiopathogenesis, risk factors, and genetic aspects. Medicina 2020 56 153. (10.3390/medicina56040153) 32244273 PMC7230892

[7] Lee WC, Kao HK, Wang SM, et al. Cold weather as a risk factor for late diagnosis and surgery for developmental dysplasia of the hip. J Bone Jt Surg Am Vol 2022 104 115–122. (10.2106/jbjs.21.00460) 34793368

[8] Meng XH, Weng YT, Rao Y, et al. Two genetic variants in the HIBCH and FTCDNL1 genes are associated with susceptibility to developmental dysplasia of the hips among the Han Chinese population of Southwest China. J Orthop Surg Res 2024 19 464. (10.1186/s13018-024-04958-8) 39113043 PMC11304665

[9] Pilz PM, Ward JE, Chang WT, et al. Large and small animal models of heart failure with reduced ejection fraction. Circ Res 2022 130 1888–1905. (10.1161/circresaha.122.320246) 35679365

[10] Willemsen K, Möring MM, Harlianto NI, et al. Comparing hip dysplasia in dogs and humans: a review. Front Vet Sci 2021 8 791434. (10.3389/fvets.2021.791434) 34977223 PMC8714762

[11] Pascual-Garrido C, Guilak F, Rai MF, et al. Canine hip dysplasia: a natural animal model for human developmental dysplasia of the hip. J Orthop Res 2018 36 1807–1817. (10.1002/jor.23828) 29227567

[12] Vanden Berg-Foels WS, Todhunter RJ, Schwager SJ, et al. Effect of early postnatal body weight on femoral head ossification onset and hip osteoarthritis in a canine model of developmental dysplasia of the hip. Pediatr Res 2006 60 549–554. (10.1203/01.pdr.0000243546.97830.a0) 16988183

[13] Roberts T & McGreevy PD. Selection for breed-specific long-bodied phenotypes is associated with increased expression of canine hip dysplasia. Vet J 2010 183 266–272. (10.1016/j.tvjl.2009.11.005) 19959383

[14] Krontveit RI, Nødtvedt A, Sævik BK, et al. A prospective study on canine hip dysplasia and growth in a cohort of four large breeds in Norway (1998–2001). Prev Vet Med 2010 97 252–263. (10.1016/j.prevetmed.2010.09.015) 20956024

[15] King MD. Etiopathogenesis of canine hip dysplasia, prevalence, and genetics. Vet Clin North Am Small Anim Pract 2017 47 753–767. (10.1016/j.cvsm.2017.03.001) 28460694

[16] Ma C, Cai G & He R. [Acetabular dysplasia: an experimental study]. Zhonghua Wai Ke Za Zhi 1998 36 559–560. 11825466

[17] Cahuzac JP, Autefage A, Fayolle P, et al. Exaggerated femoral anteversion and acetabular development: experimental study in growing dogs. J Pediatr Orthop 1989 9 163–168. (10.1097/01202412-198909020-00009) 2925850

[18] Schoenecker PL, Lesker PA & Ogata K. A dynamic canine model of experimental hip dysplasia. Gross and histological pathology, and the effect of position of immobilization on capital femoral epiphyseal blood flow. J Bone Joint Surg Am 1984 66 1281–1288. (10.2106/00004623-198466080-00018) 6490704

[19] Kasström H. Nutrition, weight gain and development of hip dysplasia. An experimental investigation in growing dogs with special reference to the effect of feeding intensity. Acta Radiol Suppl 1975 344 135–179. 1066031

[20] Smith WS, Ireton RJ & Coleman CR. Sequelae of experimental dislocation of a weight-bearing ball- and socket joint in a young growing animal; gross alterations in bone and cartilage. J Bone Joint Surg Am 1958 40-A 1121–1127. (10.2106/00004623-195840050-00015) 13587581

[21] Tachibana T, Katagiri H, Matsuda J, et al. Biomechanical analysis of load distribution in porcine hip joints at different acetabular coverages. BMC Musculoskelet Disord 2024 25 576. (10.1186/s12891-024-07701-w) 39049016 PMC11267855

[22] Alitalo I, Heikkinen E, Paatsama S, et al. Venous drainage of the femoral neck in Legg Perthes disease and in hip dysplasia. A clinical and experimental study in the dog and pig. Acta Vet Scand 1983 24 247–251. (10.1186/bf03546727) 6660161 PMC8291227

[23] Suvorov V, Filipchuk V, Mazevich V, et al. Simulation of pelvic osteotomies applied for DDH treatment in pediatric patients using piglet models. Adv Clin Exp Med 2021 30 1085–1090. (10.17219/acem/140548) 34549556

[24] Wu R, Gao G & Xu Y. Electrospun fibers immobilized with BMP-2 mediated by polydopamine combined with autogenous tendon to repair developmental dysplasia of the hip in a porcine model. Int J Nanomedicine 2020 15 6563–6577. (10.2147/ijn.s259028) 32982218 PMC7490068

[25] Gotoh E & Ando M. The pathogenesis of femoral head deformity in congenital dislocation of the hip. Experimental study of the effects of articular interpositions in pigs. Clin Orthop Relat Res 1993 288 303–309. (10.1097/00003086-199303000-00039) 8458149

[26] Yu CY, Mannen EM, Lujan TJ, et al. Porcine computational modeling to investigate developmental dysplasia of the hip. J Orthop Res 2024 42 2043–2053. (10.1002/jor.25858) 38650103

[27] Loste A, Ramos JJ, Sáez T, et al. Hip dysplasia in a 6-year-old Salz ram. Can Vet J 2003 44 140–141. 12650043 PMC340049

[28] Moraleda L, Bravo C, Forriol F, et al. Does orientation of the femoral head affect acetabular development? An experimental study in lamb. J Pediatr Orthop 2019 39 416–421. (10.1097/bpo.0000000000000974) 31393302

[29] Ooishi T. [Experimental study on the etiology of pathogenesis of acetabular dysplasia in congenital dislocation of the hip]. Nihon Seikeigeka Gakkai Zasshi 1990 64 958–975. 2266304

[30] Asplund S & Hjelmstedt A. Experimentally induced hip dislocation in vitro and in vivo. A study in newborn rabbits. Acta Orthop Scand Suppl 1983 199 1–57. (10.3109/17453678309154168) 6340409

[31] Moraleda L, Albiñana J & Forriol F. Selective epiphysiodesis of the triradiate cartilage for treatment of residual experimental acetabular dysplasia. J Pediatr Orthop 2013 33 821–828. (10.1097/BPO.0b013e31829b2f3f) 23812147

[32] Michelsson JE & Langenskiöld A. Dislocation or subluxation of the hip. Regular sequels of immobilization of the knee in extension of young rabbits. J Bone Joint Surg Am 1972 54 1177–1186. 4652049

[33] Liu X, Deng X, Ding R, et al. Chondrocyte suppression is mediated by miR-129-5p via GDF11/SMAD3 signaling in developmental dysplasia of the hip. J Orthop Res 2020 38 2559–2572. (10.1002/jor.24713) 32396235

[34] Liu J, Zhou W, Chen Y, et al. Acetabular development and fate of inverted limbus in rabbits: experimental observation from an animal model. J Orthop Res 2021 39 2595–2603. (10.1002/jor.25005) 33580529

[35] Huang SC, Liu HC & How SW. [Experimental hip dysplasia in the rabbit]. J Formos Med Assoc 1990 89 319–325. 1976751

[36] Owiny JR, Vandewoude S, Painter JT, et al. Hip dysplasia in rabbits: association with nest box flooring. Comp Med 2001 51 85–88. 11926308

[37] Gepstein R, Weiss RE & Hallel T. Acetabular dysplasia and hip dislocation after selective premature fusion of the triradiate cartilage. An experimental study in rabbits. J Bone Joint Surg Br 1984 66-B 334–336. (10.1302/0301-620x.66b3.6725340) 6725340

[38] Negri C, Tricarico A & Iorio L. The importance of the acetabular rim in the genesis of congenital hip dysplasia. Experimental research. Ital J Orthop Traumatol 1977 3 219–225. 612641

[39] Tomé I, Costa L, Alves-Pimenta S, et al. Morphometric assessment of the hip joint in a functional dysplastic rabbit model. Vet Sci 2024 11 387. (10.3390/vetsci11080387) 39195841 PMC11359858

[40] Hashimoto R, Kihara I & Otani H. Perinatal development of the rat hip joint with restrained fetal movement. Congenit Anom 2002 42 135–142. (10.1111/j.1741-4520.2002.tb00863.x) 12196711

[41] Canillas F, Delgado-Martos MJ, Martos-Rodriguez A, et al. Contribution to the initial pathodynamics of hip luxation in young rats. J Pediatr Orthop 2012 32 613–620. (10.1097/bpo.0b013e3182644948) 22892625

[42] Wang E, Liu T, Li J, et al. Does swaddling influence developmental dysplasia of the hip? An experimental study of the traditional straight-leg swaddling model in neonatal rats. J Bone Joint Surg Am 2012 94 1071–1077. (10.2106/jbjs.k.00720) 22573131

[43] Zhou W, Luo W, Liu D, et al. Fluoride increases the susceptibility of developmental dysplasia of the hip via increasing capsular laxity triggered by cell apoptosis and oxidative stress in vivo and in vitro. Ecotoxicol Environ Saf 2022 234 113408. (10.1016/j.ecoenv.2022.113408) 35298972

[44] Liu J, Bao Y, Fan J, et al. Microstructure changes and miRNA-mRNA network in a developmental dysplasia of the hip rat model. iScience 2024 27 109449. (10.1016/j.isci.2024.109449) 38551002 PMC10972838

[45] Sijbrandij S. Dislocation of the hip in young rats produced experimentally by prolonged extension. J Bone Joint Surg Br 1965 47 792–795. (10.1302/0301-620x.47b4.792) 5846782

[46] Elliott J, Ng DJ & Tham SK. Chondrocyte apoptosis in response to dislocation of the hip in the rat model. ANZ J Surg 2006 76 398–402. (10.1111/j.1445-2197.2006.03730.x) 16768703

[47] Parker MD, Clark RL, Cuttino JT Jr, et al. Streptococcal antigen-induced dislocation and dysplasia of the hip in newborn rats. Radiologic and histologic evaluation of a model of congenital dislocation of the hip. Investig Radiol 1989 24 604–608. (10.1097/00004424-198908000-00006) 2777529

[48] Yan W, Zheng L, Xu X, et al. Heterozygous LRP1 deficiency causes developmental dysplasia of the hip by impairing triradiate chondrocytes differentiation due to inhibition of autophagy. Proc Natl Acad Sci U S A 2022 119 e2203557119. (10.1073/pnas.2203557119) 36067312 PMC9477389

[49] Xu X, Bi X, Wang J, et al. Identification of KANSL1 as a novel pathogenic gene for developmental dysplasia of the hip. J Mol Med 2022 100 1159–1168. (10.1007/s00109-022-02220-4) 35727364

[50] Sun Y, You Y, Wu Q, et al. Genetically inspired organoids prevent joint degeneration and alleviate chondrocyte senescence via Col11a1-HIF1α-mediated glycolysis-OXPHOS metabolism shift. Clin Transl Med 2024 14 e1574. (10.1002/ctm2.1574) 38314968 PMC10840017

[51] Kiapour AM, Cao J, Young M, et al. The role of Gdf5 regulatory regions in development of hip morphology. PLoS One 2018 13 e0202785. (10.1371/journal.pone.0202785) 30388100 PMC6214493

[52] Feldman G, Kappes D, Mookerjee-Basu J, et al. Novel mutation in Teneurin 3 found to co-segregate in all affecteds in a multi-generation family with developmental dysplasia of the hip. J Orthop Res 2019 37 171–180. (10.1002/jor.24148) 30273960

[53] Assaraf E, Blecher R, Heinemann-Yerushalmi L, et al. Piezo2 expressed in proprioceptive neurons is essential for skeletal integrity. Nat Commun 2020 11 3168. (10.1038/s41467-020-16971-6) 32576830 PMC7311488

[54] Feldman G, Offemaria A, Sawan H, et al. A murine model for developmental dysplasia of the hip: ablation of CX3CR1 affects acetabular morphology and gait. J Transl Med 2017 15 233. (10.1186/s12967-017-1335-0) 29126427 PMC5681830

[55] Xu J, Ye W, Li H, et al. WNT1 expression influences the development of dysplasia of the hip via regulating RBPMS2/NOG-BMP2/4-GDF5- WISP2 pathway. Nucleosides Nucleotides Nucleic Acids 2022 41 765–777. (10.1080/15257770.2022.2081337) 35675541

[56] Chen Y, Li L, Wang E, et al. Abnormal expression of Pappa2 gene may indirectly affect mouse hip development through the IGF signaling pathway. Endocrine 2019 65 440–450. (10.1007/s12020-019-01975-0) 31168749

[57] Ford CA, Nowlan NC, Thomopoulos S, et al. Effects of imbalanced muscle loading on hip joint development and maturation. J Orthop Res 2017 35 1128–1136. (10.1002/jor.23361) 27391299 PMC5575772

[58] Killian ML, Locke RC, James MG, et al. Novel model for the induction of postnatal murine hip deformity. J Orthop Res 2019 37 151–160. (10.1002/jor.24146) 30259572 PMC6393179

[59] Nowlan NC, Chandaria V & Sharpe J. Immobilized chicks as a model system for early-onset developmental dysplasia of the hip. J Orthop Res 2014 32 777–785. (10.1002/jor.22606) 24590854

[60] Luo S, Chen Y, Zhou W, et al. Pioneering a chick embryo model to explore the intrauterine etiology of developmental dysplasia of the hip in oligohydramnios conditions. Osteoarthr Cartil 2024 32 869–880. (10.1016/j.joca.2024.03.118) 38588889

[61] Perry K. Feline hip dysplasia: a challenge to recognise and treat. J Feline Med Surg 2016 18 203–218. (10.1177/1098612x16631227) 26936493 PMC11148904

[62] Keller GG, Reed AL, Lattimer JC, et al. Hip dysplasia: a feline population study. Vet Radiol Ultrasound 1999 40 460–464. (10.1111/j.1740-8261.1999.tb00375.x) 10528838

[63] Chambers JN, Hanley CS & Hernandez-Divers SS. Hip luxation in a woodchuck (Marmota monax): successful treatment by closed reduction and a modified Ehmer sling. J Zoo Wildl Med 2004 35 569–571. (10.1638/04-012) 15732605

[64] Pye GW, Hamlin-Andrus C & Moll J. Hip dysplasia in koalas (Phascolarctos cinereus) at the San Diego Zoo. J Zoo Wildl Med 2008 39 61–68. (10.1638/2007-0106.1) 18432097

[65] Douglass EM. Hip dysplasia in a timber wolf. Vet Med Small Anim Clin 1981 76 401–403. 6908989

[66] Quinteros DD, García-López JM & Jenei T. Toggle-pin technique for management of coxofemoral luxation in an alpaca. Vet Surg 2011 40 369–373. (10.1111/j.1532-950x.2011.00812.x) 21361992

[67] Kierdorf U, Flohr S, Dullin C, et al. Bilateral dislocation of the hip joint and associated pathological changes in the ossa coxae and femora of a European roe deer (Capreolus capreolus). PLoS One 2023 18 e0290586. (10.1371/journal.pone.0290586) 37616270 PMC10449113

[68] Balisi MA, Sharma AK, Howard CM, et al. Computed tomography reveals hip dysplasia in the extinct Pleistocene saber-tooth cat Smilodon. Sci Rep 2021 11 21271. (10.1038/s41598-021-99853-1) 34711910 PMC8553773

[69] Mostafa AA, Berry CR & Nahla MA. Quantitative assessment of hip morphology to enhance the identification of hip dysplasia in German Shepherd Dogs. Am J Vet Res 2023 84 1–10. (10.2460/ajvr.22.09.0165) 36729899

[70] Mostafa AA, Nahla MA, Ali KM, et al. Modified FCI (fédération cynologique internationale) scoring of the coxofemoral joint in labrador Retrievers without and with hip dysplasia. Front Vet Sci 2022 9 800237. (10.3389/fvets.2022.800237) 35372531 PMC8971752

[71] Fu M, Liu J, Huang G, et al. Impaired ossification coupled with accelerated cartilage degeneration in developmental dysplasia of the hip: evidences from μCT arthrography in a rat model. BMC Musculoskelet Disord 2014 15 339. (10.1186/1471-2474-15-339) 25294293 PMC4289046

[72] Zhang H, Wang L, Cui J, et al. Maintaining hypoxia environment of subchondral bone alleviates osteoarthritis progression. Sci Adv 2023 9 eabo7868. (10.1126/sciadv.abo7868) 37018403 PMC10075992

[73] Carneiro RK, da Cruz ICK, Gasser B, et al. B-mode ultrasonography and ARFI elastography of articular and peri-articular structures of the hip joint in non-dysplastic and dysplastic dogs as confirmed by radiographic examination. BMC Vet Res 2023 19 181. (10.1186/s12917-023-03753-7) 37784120 PMC10544497

[74] Xu Y, Tian NX, Bai QY, et al. Gait assessment of pain and analgesics: comparison of the DigiGait™ and CatWalk™ gait imaging systems. Neurosci Bull 2019 35 401–418. (10.1007/s12264-018-00331-y) 30659524 PMC6527535

[75] Çağlar C, Kara H, Ateş O, et al. Evaluation of different intraarticular injection therapies with gait analysis in a rat osteoarthritis model. Cartilage 2021 13 (Supplement 2) 1134s–1143s. (10.1177/19476035211046042) 34528494 PMC8804824

[76] Marinou KA & Dontas IA. European union legislation for the welfare of animals used for scientific purposes: areas identified for further discussion. Anim 2023 13 2367. (10.3390/ani13142367) PMC10376073 37508144

[77] Park YS, Konge L & Artino AR Jr. The positivism paradigm of research. Acad Med 2020 95 690–694. (10.1097/ACM.0000000000003093) 31789841

[78] National Research Council (US) Institute for Laboratory Animal Research. Guide For the Care and Use of Laboratory Animals, 8th edn. Washington, DC: National Academies Press (US). (https://www.ncbi.nlm.nih.gov/books/NBK232589/) 25121211

[79] Kilkenny C, Browne WJ, Cuthill IC, et al. Improving bioscience research reporting: the ARRIVE guidelines for reporting animal research. PLoS Biol 2010 8 e1000412. (10.1371/journal.pbio.1000412) 20613859 PMC2893951

